# Pregnancy and diabetic ketoacidosis: fetal jeopardy and windows of opportunity

**DOI:** 10.3389/fcdhc.2023.1266017

**Published:** 2023-11-16

**Authors:** Ankia Coetzee, David R. Hall, Eduard J. Langenegger, Mari van de Vyver, Magda Conradie

**Affiliations:** ^1^ Department of Medicine, Division of Endocrinology Stellenbosch University and Tygerberg Hospital, Cape Town, South Africa; ^2^ Department of Obstetrics and Gynecology, Stellenbosch University and Tygerberg Hospital, Cape Town, South Africa; ^3^ Department of Medicine, Division of Clinical Pharmacology, Stellenbosch University and Tygerberg Hospital, Cape Town, South Africa

**Keywords:** DKA, hyperglycemia in pregnancy, gestational diabetes mellitus, HbA1c, hypokalemia

## Abstract

**Background:**

Diabetic ketoacidosis (DKA) during pregnancy poses significant risks to both the mother and fetus, with an increased risk of fetal demise. Although more prevalent in women with Type I diabetes (T1D); those with Type 2 diabetes (T2D) and gestational diabetes mellitus (GDM) can also develop DKA. A lack of information about DKA during pregnancy exists worldwide, including in South Africa.

**Objective:**

This study examined the characteristics and outcomes associated with DKA during pregnancy.

**Methods:**

The study took place between 1 April 2020 and 1 October 2022. Pregnant women with DKA, admitted to Tygerberg Hospital’s Obstetric Critical Care Unit (OCCU) were included. Maternal characteristics, precipitants of DKA, adverse events during treatment, and maternal-fetal outcomes were examined.

**Results:**

There were 54 episodes of DKA among 47 women. Most DKA’s were mild and occurred in the third trimester. Pregestational diabetes dominated (31/47; 60%), with 47% having T1D and 94% requiring insulin. Seven women (7/47, 15%; T2D:6, T1D:1) had two episodes of DKA during the same pregnancy. Most women (32/47; 68%) were either overweight or obese. Yet, despite the T2D phenotype, biomarkers indicated that auto-immune diabetes was prevalent among women without any prior history of T1D (6/21; 29%). Twelve women (26%) developed gestational hypertension during pregnancy, and 17 (36%) pre-eclampsia. Precipitating causes of DKA included infection (14/54; 26%), insulin disruption (14/54; 26%) and betamethasone administration (10/54; 19%). More than half of the episodes of DKA involved hypokalemia (35/54, 65%) that was associated with fetal death (P=0.042) and hypoglycemia (28/54, 52%). Preterm birth (<37 weeks’ gestation) occurred in 85% of women. No maternal deaths were recorded. A high fetal mortality rate (13/47; 28%) that included 11 spontaneous intrauterine deaths and two medical terminations, was observed.

**Conclusion:**

Women with DKA have a high risk of fetal mortality as well as undiagnosed auto-immune diabetes. There is a strong link between maternal hypokalemia and fetal loss, suggesting an opportunity to address management gaps in pregnant women with DKA.

## Introduction

The prevalence of Type 2 diabetes mellitus (T2D) has been rising more rapidly in developing countries, and its age of onset is decreasing (1). Women of reproductive age are thereby also affected (1–3). Any form of diabetes can overlap with pregnancy, but gestational diabetes mellitus (GDM) occurs exclusively during pregnancy (4–6). Although diabetic ketoacidosis (DKA) is traditionally believed to be a complication of Type 1 diabetes mellitus (T1D), it is increasingly being reported in people with T2D and GDM (7, 8). DKA in pregnancy is associated with maternofetal morbidity and a high fetal mortality rate (9–11).

Numerous physiological adaptations during pregnancy predispose to DKA, resulting in a higher rate of DKA compared to non-pregnancy (8). The placenta secretes several diabetogenic hormones that are responsible for increased maternal insulin resistance (IR). This leads to increased gluconeogenesis, glycogenolysis, and lipolysis. Lower fasting glucose levels further promote lipolysis and ketogenesis. Lipolysis generates free fatty acids, increases acidic metabolites, and yields ketones as an alternative energy source. The physiological adaptation of accelerated starvation contributes to ketosis. Aside from these changes, the maternal capacity for buffering acid decreases due to an increase in alveolar ventilation and a decline in bicarbonate levels (8, 12, 13).

The combination of hyperglycemia, ketonemia and a high anion gap metabolic acidosis suggests the presence of DKA (7). Hyperglycemia during DKA in pregnancy may be less pronounced and thus result in late diagnoses. This is due to lower fasting glucose levels, increased glomerular filtration, and trans-placental glucose transport.

In addition to the inherent higher background risk for DKA in pregnancy, commonly used drugs such as glucocorticoids and pro-adrenergics have also been linked to DKA (12, 14, 15). Infections, the lack of access to health care, and delayed diagnoses pose as risk factors for DKA in developing countries (16–18). The interaction of these factors may contribute to maternofetal adversity and add to the healthcare burden associated with DKA in pregnancy. Published data on the adversity and impact of DKA in pregnancy is limited despite its relatively common occurrence. The aim of this study was to describe DKA events in pregnant women admitted to the inpatient obstetric facility at Tygerberg Hospital, and to examine the associated clinical risk factors and pregnancy outcomes.

## Methods

### Study population

A descriptive study based on retrospective data collection was conducted in the OCCU at Tygerberg Hospital. Tygerberg is a tertiary public health care hospital in Cape Town, South Africa, affiliated to the University of Stellenbosch. All pregnant women admitted with confirmed DKA to the OCCU for the study period April 2020 to October 2022 were eligible for study entry. The criteria for DKA diagnosis were a pH ≤ 7.30; bicarbonate level ≤ 18 mEq/L and blood glucose >10mmol/l (19, 20). Women with euglycemic DKA were also included (see clarification in DKA characteristics section below). Pregnant women with DKA are managed in accordance with standard institutional protocols.

### Data collection

Patient clinical information and data were retrieved from the Tygerberg Electronic Content Management System (ECM) and biochemical results obtained via the National Health Laboratory System (NHLS) electronic platform. Strict confidentiality was maintained as the ECM and NHLS databases, as well as personal computers used for data collection, are password protected thereby restricting access to data. Patient data, including the medical and antenatal history, socio-demographic characteristics, clinical findings, and laboratory parameters during the acute presentation and at delivery were entered onto a spreadsheet using Excel version 2019 Microsoft Office Professional Plus (Microsoft Corp, Redmond, WA, USA). A unique number was linked to each patient’s data set to de-identify the patient.

### Maternal demographics, anthropometry, and clinical outcome measures

Demographic data pertaining to maternal age (years), parity, gestational age (weeks) and anthropometric measurements [weight (kg) and height (m)] at the first antenatal visit for the corresponding pregnancy were recorded. Body mass index (BMI) (kg/m^2^) values were calculated and divided into weight categories according to the World Health Organisation’s (WHO) classification (21). Information regarding social stressors and the use of illicit substances was collected and recorded.

The presence of either known pregestational diabetes prior to the index pregnancy or hyperglycemia first diagnosed during pregnancy was sought and noted. The duration of the condition, the historic type assigned (T1D or T2D) and antenatal diabetes medication was documented in those with known diabetes. The total daily dose (IU) of prescribed and required insulin pre-DKA admission and after discharge were calculated and compared.

Data on blood pressure (BP), the use of antihypertensive medication, evidence of pre-eclampsia with loss of blood pressure control with or without development or worsening of proteinuria in pregnancy were retrieved and documented. Study participants were classified with either chronic hypertension, new onset gestational hypertension or suspected or confirmed new onset or superimposed pre-eclampsia. Gestational hypertension was defined as a systolic blood pressure (SBP) ≥140 mm Hg and/or a diastolic blood pressure (DBP) ≥90 mm Hg on two occasions, four hours apart, after 20 weeks of gestation, in a woman with previously normal BP (22). A new diagnosis of preeclampsia was made in the presence of new onset hypertension after 20 weeks’ gestation in combination with newly developed proteinuria (≥300 mg per 24-hour urine collection) and/or significant end-organ dysfunction. A SBP ≥140 mmHg or a DBP ≥ 90mm Hg on two occasions, four hours apart, in a previously normotensive individual, or a single confirmed SBP ≥160 mmHg or DBP ≥ 110mm Hg served as BP criteria for new onset pre-eclampsia. In the absence of proteinuria, preeclampsia was also considered in the presence of hypertension criteria as mentioned along with concurrent thrombocytopenia and evidence of functional impairment of end-organs in accordance with the diagnostic criteria proposed by the (the International Society for the study of Hypertension in Pregnancy) (22). The term superimposed preeclampsia was used for women with known chronic hypertension presenting with loss of BP control or resistant hypertension (especially acutely), worsening proteinuria, and/or end-organ dysfunction after 20 weeks of gestation.

### Characteristics of diabetic ketoacidosis events

The characteristics of all DKA events were documented and described for the whole study cohort and stratified based on the severity of the DKA episode. The severity of DKA was stratified on pH (mild: pH 7.25-7.30; moderate: pH 7.0-7.24; severe: pH <7.0) A diagnosis of euglycemic DKA was made when glucose levels were <11.1 mmol/l, urine ketones were present, and other causes of metabolic acidosis excluded (23–25). When pregnant women present with DKA, it is expected of the attending clinician to test for antibodies to glutamic acid decarboxylase (anti-GAD antibodies) to identify or exclude auto-immune-mediated diabetes if the diagnosis is not already known.

Data on precipitants of the event, the timing in pregnancy (gestational age), biochemical parameters, the occurrence of adverse events, the required duration of stay in a critical care unit and standard management pertaining to fluid administration and insulin therapy was captured. The number of all maternal hypoglycemic (glucose <4 mmol/l) and hypokalemic (potassium <3.5 mmol/l) episodes were closely monitored and recorded in the first 24 hours post-admission as part of standard practice. Data on fluid and insulin requirements, the transition process from intravenous to subcutaneous insulin and the time to the resolution of each DKA episode (pH >7.30, bicarbonate level >18) was captured.

Biochemistry was performed at the NHLS, a lab accredited by the South African National Accreditation System (SANAS) at Tygerberg Hospital. Electrolytes were measured using Roche Cobas^®^ 6000 (Roche Diagnostics, Germany). To identify auto-immune diabetes cases, an ELISA kit manufactured by EUROIMMUN Medizinische Labordiagnostika AG (Germany) was used for anti-GAD testing (sensitivity: 96%, specificity: 98%) (26). Islet Autoantibody Standardization Program used human recombinant glutamic acid decarboxylase isoform GAD65 for coating and preparing biotinylated GAD. The lower detection limit of the Anti-GAD ELISA was 0.59 IU/ml. As per EUROIMMUN recommendations, results were interpreted as follows: 0-10 IU/ml: negative, and ≥10 IU/ml: positive (27).

### Delivery information

Delivery information obtained included the timing between the DKA event and delivery (days), the gestational age (weeks), the mode of delivery, and the birth weight (g). The timing between the DKA event and delivery was based on the most recent DKA episode in women who have had more than one episode. Gestational age was based on ultrasound ± last menstrual period. Early ultrasound was defined as less than 24 weeks of pregnancy, according to local practice. This differs from international terminology that defines an early ultrasound as a study conducted before 12-14 weeks of pregnancy (28–30). Preterm delivery referred to a delivery before 37 weeks of gestation and macrosomia was defined as a birth weight more than 4000 g (30). Fetal loss included spontaneous intrauterine death (IUD), medical termination and non-viable early perinatal death. The definition of stillbirth was based on WHO criteria (birth at ≥20 weeks of gestation with no signs of life) (31).

### Statistical analysis

GraphPad Prism (9.5.1) was used for data analysis. Descriptive statistics were used for socio-demographic parameters and reported as number (%). The distribution of data was assessed using the Shapiro-Wilk and Kolmogorov-Smirnov normality tests. Normally distributed data are reported as mean ± standard deviation (SD) and non-parametric data as median and interquartile range (IQR). Continuous variables were compared using either the t test (for normally distributed variables) or Mann–Whitney U test (non-parametric data). Receiver Operator Curves (ROC) and Kaplan Meier Survival curve analysis was performed to assess associations between variables and outcomes. Fisher’s exact tests were used to assess associations across demographic and clinical characteristics of women with DKA. The threshold level for significance was accepted at p<0.05.

Informed consent was waived due to the retrospective nature of data collection. The study complied with the World Medical Association Declaration of Helsinki and was approved by the Health Research Ethics Committee (HREC) of the Faculty of Medicine & Health Sciences, Stellenbosch University (S21/11/255).

## Results

### Maternal characteristics

**Figure 1:**
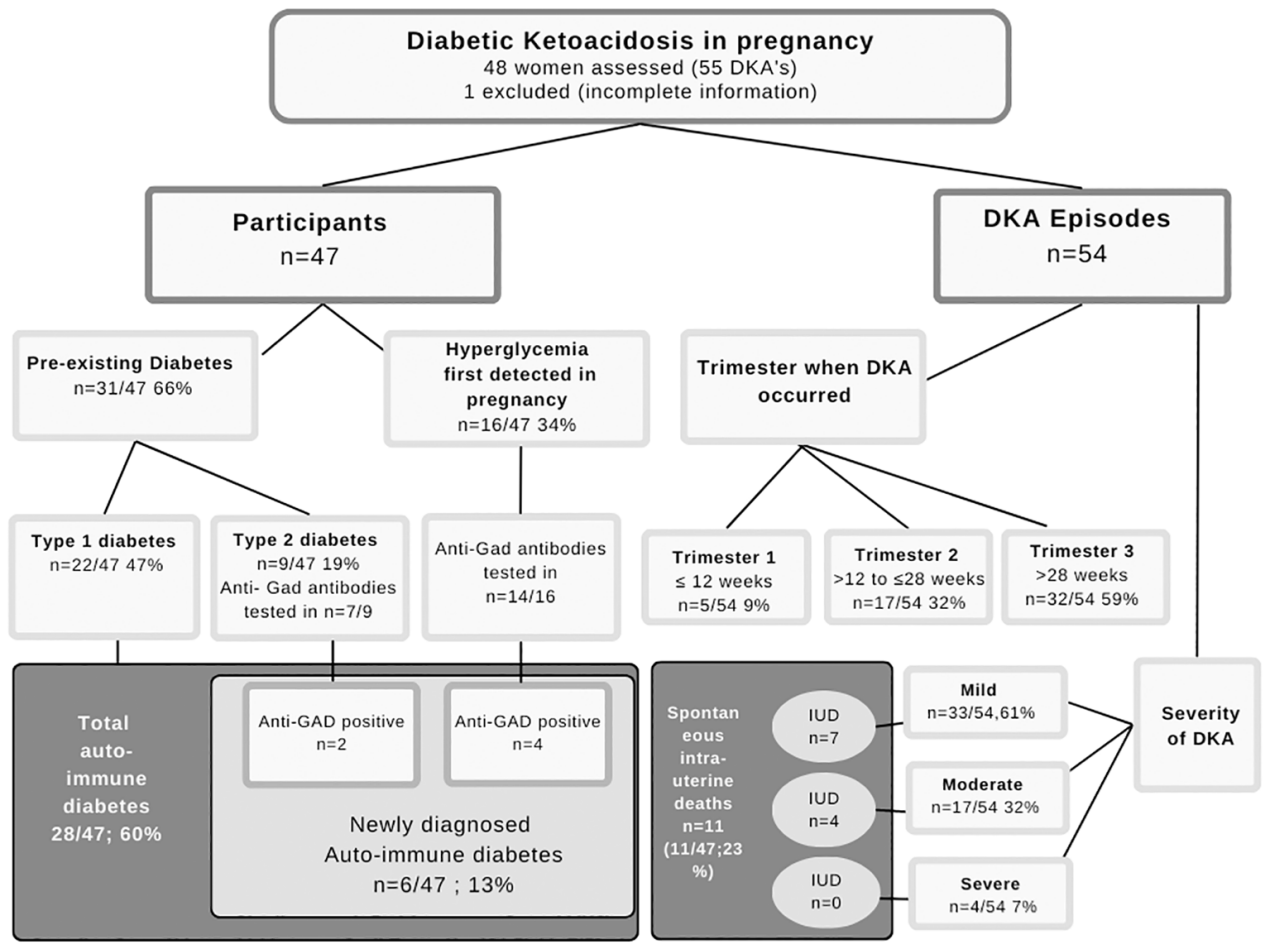
Diabetes type and DKA occurrence.

Forty-seven women, all with singleton pregnancies, had a total of 54 confirmed DKA episodes that were treated in the Obstetric Critical Care Unit (OCCU) during the reporting period. The study population consisted of a diverse population representative of the demographics in the Western Cape Provence. An overview is presented in **Figure 1**. Of these, 15 (34%) had suffered a fetal loss in a prior pregnancy. More than one DKA episode was recorded in seven women (7/47;15%). Maternal characteristics for all pregnancies complicated by DKA and stratified based on birth outcome (live births, fetal loss) are summarized in **Table 1**. The mean maternal age in the total cohort was 27 years. At the time of the first prenatal visit, the group’s median BMI was within the overweight WHO category (28kg/m^2^, IQR 23-32). Sixty eight percent of mothers were either overweight or obese and included 13 of the 22 mothers with T1D. Most women booked early in pregnancy, 21 attended for their first antenatal visit before 14 weeks and only seven women (11%) postponed antenatal care till after 24 weeks.

**Table 1:** Maternal characteristics stratified by fetal outcome.

	All	Live births	All fetal losses^*^	p-value
	N=47	N=34 (72%)	N=13 (28%)	
Total number of DKA events	54	39 (72%)	15 (28%)	
Pregnancies with multiple DKAs	7 (15%)	5 (15%)	2 (15%)	NS
Age (yrs)	27 ± 5	28 ± 5	26 ± 5	NS
≥35 yrs	5 (11%)	5 (15%)	0	SS
Gravidity (n)	2 (1-3)	3 (1-3)	2 (1-3)	NS
Primigravida’s	17 (56%)	11 (32%)	6 (46%)	NS
Parity (n)	1 (0-2)	1 (0-2)	1 (0-1)	NS
Weight at booking visit (kg)	70 (61-82)	71 (63-82)	62 (55-81)	NS
BMI^**^ at booking visit (kg/m^2^)	28 (23-32)	28 (25-32)	26 (21-32)	NS
Overweight and obese(BMI>24.9kg/m^2^) n(%)	32(68%)	24(71%)	6 (46%)	SS
Gestation at booking visit (wks)	13 (8-19)	13 (8-19)	13 (8-19)	SS
Pregestational hypertension n(%)	22 (47%)	17 (50%)	5 (39%)	SS
Diabetes (pre-existing)	31 (66%)	24 (71%)	7 (54%)	NS
Type 1	22 (47%)	16 (47%)	6 (46%)	SS
Type 2	9 (19%)	8 (24%)	1 (8%)	SS
Diabetes first diagnosed in pregnancy	16 (34%)	12 (35%)	4 (31%)	NS
Before DKA event	5 (11%)	5(15%)	0	SS
At DKA event	11(23%)	7 (21%)	4 (31%)	SS
Treatment at DKA event				
Insulin	29/31 (94%)	22(65%)	7(54%)	NS
Metformin	10 (21%)	8 (24%)	2 (15%)	SS
None (new diagnosis)	16 (34%)	10 (29%)	6 (46%)	NS
Duration (yrs)	6.5 (3-13.5)	7 (3-15)	5 (2-16)	NS
Maternal glycemic control:				
pre-DKA HbA1c (%)^#^	10.4 (7.7-11.9)	10.3 (7.7-12.1)	10.6 (7.3-11.7)	NS
Mmol/mol	90 (61-107)	89(61-109)	92(56-104)	

DKA, Diabetic ketoacidosis; BMI, Body mass index, *Spontaneous deaths and medical terminations. **Weight categorization according to World Health Organization. # Sixteen women with pre-existing diabetes who had an HbA1c in this pregnancy.

NS, non-significant i.e. P>0.05 SS, Sample size too small.

Type 1 diabetes was present in 47% and T2D in 19% of cases. In the pregnancies complicated by DKA, a new diagnosis of hyperglycemia first detected in pregnancy (HFDP) was made in 34% (16/47). A total of 11 of the newly diagnosed cases were detected when the patient presented with DKA. Insulin therapy was required for glycemic control in 29 of 31 women with pre-existing diabetes, with a duration of 6.5 (IQR 3.0-13.5) years. Sixteen women with pre-existing diabetes had an HbA1c in this pregnancy with a median HbA1c of 10.4% (IQR 7.7-11.9). The poor control was similar across severity subgroups.

Anti-GAD antibody results were available in 21 women not defined as having T1D (14/16 women with HFDP and 7/9 with pre-existing T2D). In six women with DKA, immune-mediated T1D was diagnosed following a positive anti-GAD antibody test. Four of these women presented with HFDP, while two were previously diagnosed with T2D. Both cases with a prior diagnosis of T2D were 35 years of age, in the WHO overweight category (BMI 25.7kg/m^2^ and 25.8kg/m^2^) and were receiving insulin. They were known with diabetes for three and nine years respectively.

All seven women who presented with multiple DKAs (7/47; 15%) had pre-existing diabetes mellitus (T1D:6 and T2D:1). Four women admitted to the use of illicit substances during pregnancy, whereas significant social stressors, such as food insecurity, was experienced by nine women.

### Characteristics of diabetic keto-acidosis events

**Table 2** summarizes the characteristics of DKA events according to the severity classification at presentation (mild, moderate, severe). The majority of DKA episodes (61%) were mild.

**Table 2:** Characteristics of DKA episodes stratified by severity.

	Total episodes (N =54)	Mild (N=33)	Moderate (N=17)	Severe (N=4)
Biochemistry at presentation				
Acid base				
pH	7.27 (7.16-7.30)	7.29 (7.28-7.3)	7.14 (7.13-7.22)	6.90 (6.91-6.93)
HCO3^-^ (mmol/l)	10.06 ± 4.15	12.33 ± 2.69	7.59 ± 3.15	2.4 ± 1.7
Base excess(mmol/l)	14.8 (12.0-18.9)	13.4 (10.0-15.0)	20.5 (16.3-23.5)	26 (25-27)
Lactate(mmol/l)	1.1 (0.7-1.5)	1 (0.7-1.35)	1.3 (1.1-2.3)	1.3 (1.2-2.8)
Glycemic status and control				
Blood glucose (mmol/l)	19.0 ± 5.1	18.3 ± 4.6	19.4 ± 5.6	23.4 ± 4.7
HbA1c (%)	9.2 (7.7-11.3)	9.8 (7.7-11.3)	8.8 (6.9-11.2)	8.9 (8.5-11.6)
HbA1c (mmol/mol)	77 (61-100)	84 (61-100)	73 (52-99)	74 (69-103)
Potassium on presentation (mmol/l)	4.1 (3.6-4.5)	3.8 (3.4-4.4)	4.3 (4.2-4.6)	4.5 (4.0-5.9)
Hypokalemia (K<3.5mmol/l) n(%)	7 (13%)			
Infective markers				
C-reactive protein mg/l	5 (25-94)	24 (6-172)	42 (6-89)	22 (5-68)
**Gestation at DKA event n=54** (weeks)	29 (26-32)	29 (23-32)	31 (27-35)	26 (11-27)
Precipitant^*^ n(%)				
More than one precipitant per DKA episode	2 (4%)	1(3%)	0	1 (25%)
Infection n(%)	14 (26%)	9 (27%)	3 (18%)	2 (50%)^$^
Urinary tract infection n(%)^**^	11/14 (79%)	9/9 (100%)	2/3 (67%)	0
Insulin discontinued n(%)	14 (26%)	9 (27%)	2 (12%)	3 (75%)
Betamethasone n(%)	11 (20%)	7 (21%)	4 (24%)	0
Vomiting n(%)	3 (6%)	2 (6%)	1 (6%)	0
No precipitant identified n(%)	12(22%)	5(18%)	7(41%)	0
Adverse event in first 24-hours after insulin initiation				
Hypokalemia (K<3.5mmol/l) n(%)	35 (65%)	20 (61%)	12 (71%)	3 (75%)
Hypoglycemia (<4.0mmol/l) n(%)	28 (52%)	18 (55%)	7 (41%)	3 (75%)
>1 episode of hypoglycemia n(%)	14 (26%)	8 (24%)	5 (29%)	1 (25%)
Hypoglycemia and hypokalemia n(%)	17 (31%)	9 (27%)	5 (29%)	3 (75%)
Management				
Total length of hospital stay (days)	7.5 (6.0-11.0)	7.0 (5.5-11.0)	8.0 (6.0-12.0)	8.5 (5.8-11.3)
Length of stay in OCCU (days)	3 (2-4)	3 (2-4)	3 (3-5)	4.5 (2.5-5)
Resolution of individual parameters of event in hours				
pH >7.30 (hrs)	15.5 ± 10.9	12.9 ± 11.6	19.1 ± 8.8	21.3 ± 8.9
HC03^-^ >15 mmol/l (hrs)	25.7 ± 16.0	20.8 ± 16.3	33.1 ± 13.6	32.0 ± 10.8
Glucose <12 mmol/l (hrs)	5.4 ± 3.9	4.7 ± 2.8	6.5 ± 5.6	6.3 ± 1.3
IV fluid^*^ administered (ml/24hrs)	4126 ± 1258	4040 ± 1188	4142 ± 1436	4765 ± 1137
Potassium administered (mmol/24hours)	80 (40-120)	40 (25-80)	90 (80-120)	120 (80-120)
Insulin (IU/24hrs)	68 (42-114)	66 (41-91)	112 (48-125)	94 (66-129)

OCCU, obstetric critical care unit; *includes DKA events where more than one precipitant occurred **urinary tract infections expressed as percentage of total number of infections recorded as precipitants.

^$^Two different infections occurred in a single patient.

The overall HbA1c was high at DKA presentation (HbA1c: 9.2% (7.7-11.3); 77mmol/mol (61–100) denoting poor glucose control.

Euglycemic DKA was documented in two cases, one mild and one moderate event. The mild event occurred as a second DKA in the same pregnancy. Hyperglycemia associated with keto-acidosis was obscured due to self-administered insulin corrections just prior to presentation (recorded blood glucose on admission of 7.8 mmol/L). The diagnostic blood glucose in the moderate event was 10.5 mmol/L in the presence of a metabolic acidosis (pH 7.17, bicarbonate 8 mEq/L) and after exclusion of alternate causes. DKA events occurred mostly early in the third trimester of pregnancy (median gestation 29 weeks IQR 26-32).

At the time of admission, potassium levels inversely correlated with the severity of DKA (R2 = 0.09, p=0.03), with the highest levels, as expected, documented for the severe DKA events. Hypokalemia (potassium level of <3.5 mmol/l) was present at baseline prior to any intervention in seven DKA events.

Infections, especially urinary tract infections (11/14; 79% of all infections) and discontinuation of insulin therapy were the two most common precipitating factors. Further, a Covid-19 pneumonia, an influenza A infection, and a breast abscess were recorded. In most cases (42/54; 78%) at least one precipitating factor was present (see **Table 2**). Twenty percent (11/54) of all DKA events followed the administration of betamethasone, in eight of these cases the betamethasone was given at the primary care facility prior to referral. Vomiting was a presenting feature in three women at presentation. After initiation of the standard OCCU DKA therapy protocol, hypokalemia was noted in most episodes (34/54, 65%).

Over half (52%) of the DKA events also involved hypoglycemia during treatment, with 26% having multiple episodes. Neither the baseline blood glucose on admission (19 mmol/L (13.4-23.9IQR) vs 16.1 mmol/L (12.1-20.2 IQR) p=0.1392) nor the total dosage of administered insulin in the first 24 hrs (96 IU (42-120 IQR) vs 67 IU (34-102 IQR) p=0.2313) were significantly associated with the development of hypoglycemia. There were 17 events (31%) complicated by both hypokalemia and hypoglycemia.

A total of 175 of the 484 admission days for DKA were spent in the high care unit (OCCU), Time spent in the OCCU were three days (2-4 IQR), whilst 7.5 days (6-11 IQR) was spent in hospital overall. In the first 24 hours of hospitalization, approximately four liters of intravenous fluid were administered to each patient with DKA. Potassium replacement were 80 (40-120 IQR) mmol per DKA event, but dose requirements increased with the severity. With DKA resolution, the insulin infusions were overlapped with subcutaneous short- or intermediate-acting insulin in most (50/54; 93%).

### Pregnancy outcome

Pregnancy outcomes are tabulated in **Table 3**. The gestational age at delivery was 34 weeks (IQR 31-36) with 85% of deliveries preterm and 55% coinciding with a DKA admission. Among the 34 live births, preterm births accounted for 79%. A total of 13 (28%) fetal losses occurred. It included nine stillbirths, one miscarriage and two medical terminations of pregnancy, respectively at 12 and 30 weeks of gestation. The late termination at 30 weeks was fetocide for several congenital anomalies. There was a similar percentage of spontaneous intrauterine deaths and stillbirths among pregnancies complicated by mild and moderate DKA events (7/33 (20%) and 4/17 (24%), respectively). There were no fetal losses in the four pregnancies complicated by severe DKA events. Two of the 11 spontaneous intrauterine deaths occurred in pregnancies complicated by multiple DKA episodes. There were no maternal deaths.

**Table 3:** Pregnancies complicated by DKA events in total cohort and stratified by fetal outcome.

	Total N=47	Live birth N=34 (72%)	Fetal loss N=13 (28%)	P=value
Gestation at delivery in weeks	34 (31-36)	34 (32-36)	32 (30-35)	NS
Preterm deliveries (<37 wks) n (%)	40 (85%)	27 (79%)	13 (100%)	NS
Delivery with DKA event n (%)	26 (55%)	17 (50%)	9 (69%)	NS
Days from DKA to delivery	14 (2-42)	24 (3-46)	2 (1-28)	NS
Birth outcome				
Miscarriage n(%)	1 (2%)		1(8%)	SS
Stillbirth n(%)	9 (19%)		9 (69%)	SS
Termination n(%)	2(4%)	0	2 (16%)	SS
Late termination (>20 weeks)	1(2%)		1 (8%)	SS
Maternal mortality n(%)	0	0	0	
Gestational hypertension n(%)	10 (21%)	8 (24%)	2 (15%)	SS
Pre-eclampsia n(%)	17 (36%)	13 (38%)	4 (31%)	SS
Mode of delivery				
Vaginal delivery	20	8 (24%)	12 (92%)	P<0.01
Induced labor n(%)	14 (30%)	10 (29%)	4 (31%)	SS
Caesarean section n(%)	27 (57%)	26 (76%)	1 (8%)	SS
In live births				
Birthweight (gram)	2523 (1490-3163)	2600 (1465-3270)	1820 (1705-3080)	NS
Macrosomia (>4000gram) n(%)		1 (3%)		SS

DKA, Diabetic ketoacidosis; SS, sample size too small; NS, Not significant.

A large percentage of women in this cohort had hypertensive conditions, including 22(47%) with pregestational hypertension and 10 (21%) with gestational hypertension. Pre-eclampsia was identified in 17 (36%) of the cohort. The incidence of preeclampsia was 36% (8 out of 22) in women with pregestational hypertension and 80% (8 out of 10) in women with gestational onset hypertension respectively.

**Figure 2:**
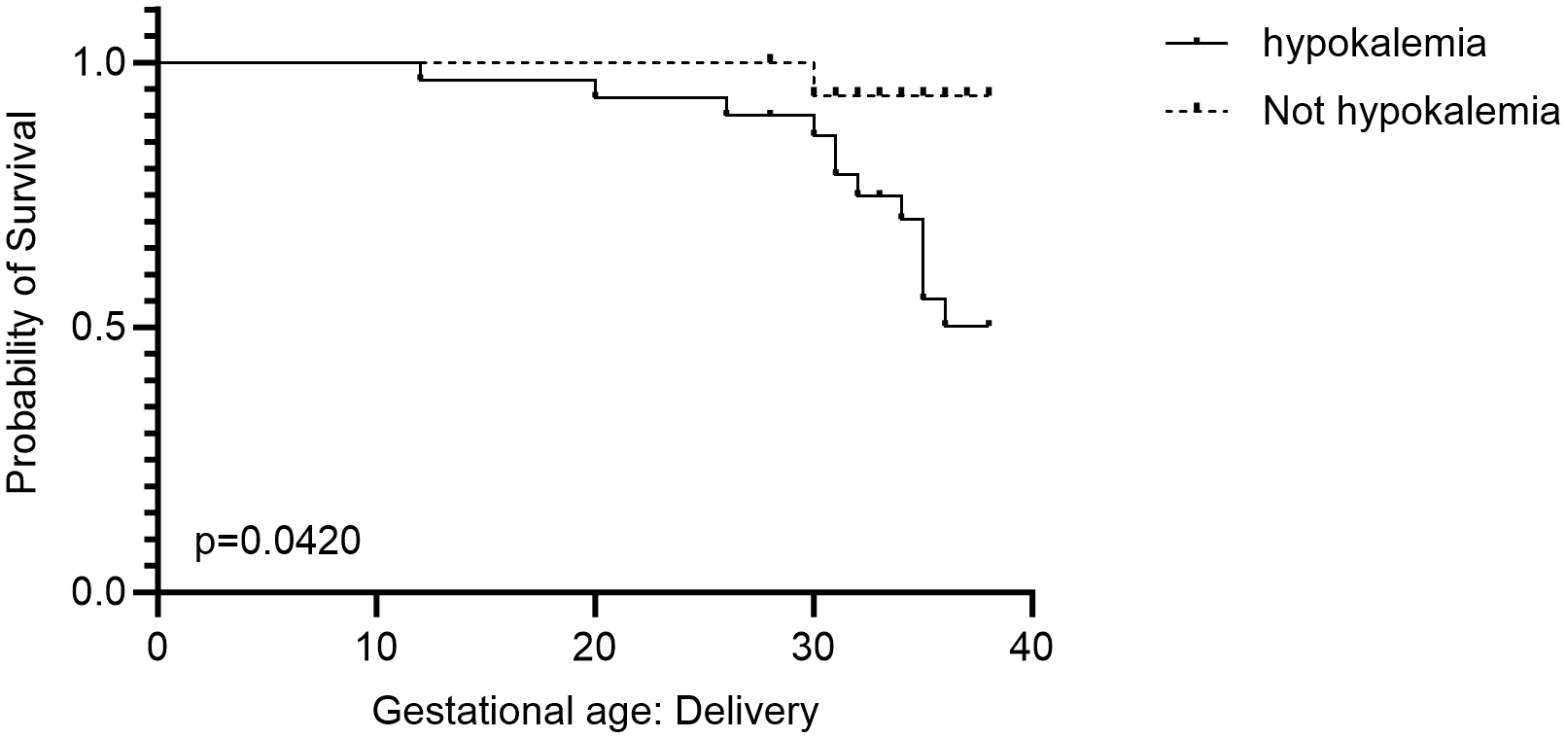
Kaplan Meier Survival curve depicting the relationship between fetal loss and hypokalemia.

Six of the fetal losses (6/13; 46%) occurred in women with hypertensive disorders; four of these (4/13; 31%) had pregestational hypertension. The other two cases were in women with gestational hypertension who subsequently developed preeclampsia. With the exception of one case (12/13,92%), all women with fetal losses had iatrogenic hypokalemia and five had concurrent hypoglycemia within the first 24 hours of admission. There was a significant association between hypokalemia and fetal loss (p =0.042) depicted in **Figure 2**.

## Discussion

Our study reports on 54 DKA events in 47 pregnancies observed over a period of 31 months in a single tertiary care center in South Africa. Despite substantial efforts to improve antenatal screening and optimize the management of pregnancy-related hyperglycemia, a high number of DKA events are still encountered in our institution. There were no maternal deaths, but morbidity was high, with an estimated 68% of women diagnosed with chronic hypertension, gestational hypertension or pre-eclampsia. The number of pregnancies with fetal loss was higher than figures reported from high-income countries (10, 32).. Most DKA events were mild and occurred in the third trimester of pregnancy in women with T1D. Almost one quarter (23%) of DKA women had diabetes diagnosed during DKA in pregnancy episode. Events were mainly precipitated by infection, non-adherence to medication and steroid therapy. Only two of 54 DKA’s were euglycemic, and 15% of women had more than one DKA in the index pregnancy.

Published data on DKA in pregnancy are limited and mostly represented by case reports, case series or small cohorts in high income countries (9, 10, 32–35). In the literature, most DKA cases are mild and occur late in pregnancy, findings consistent with ours (10, 14, 15, 32, 35, 36). Two of the larger case series of pregnancy-related DKA are Dhanasekaran et al.’s single- center study performed in Rochester, Minnesota and Diguisto et al.’s multi-center study from the United Kingdom (UK) (32, 37). Dhanasekaran et al., examined 71 DKA events that took place over 17 years, which was considerably longer than our study period, whereas Diguisto et al.’s reported on 83 DKA cases over a 22-month period (32, 37). Thus the number of DKA’s reported in our study appears to be high. Variables impacting on DKA risk are not uniform in different geographical regions. Differences in healthcare access, referral pathways, time to presentation, antenatal screening practices, and socioeconomic status all contribute to the varied number of DKA events reported in the literature.

Diabetes is presently one of the most commonly diagnosed diseases in South Africa, with a tripling in the number of new cases between the years 2010 and 2019 (38). The high background prevalence of diabetes, poor socioeconomic conditions, and limited access to formal health care all contribute to the risk of DKA nationally. In this study the main precipitants of DKA in this population of pregnant women were infectious conditions, especially of the urinary tract, non-adherence to insulin therapy, and steroid therapy. Only three women (6%) reported vomiting. These precipitating factors are in accordance with available literature and noted by most researchers to be the main contributors (10, 15, 32, 34, 37). Dhanasekaran et al., noted higher rates of non-adherence amongst women with DKA events than in our study (40 vs 23%), but report a similar rate of infection (22%) (37). The study by Diguisto et al., indicated that infections and vomiting were the most common precipitating factors in their UK cohort (32).

Like other published studies, the majority of women in our study of DKA events had T1D. The study included 47 participants, 22 of whom had previously been diagnosed with T1D, with an additional diagnosis of immune-mediated T1D made in six women at the time of the DKA event. Management of T1D in pregnancy is challenging for both patients and care providers. Near-normalization of maternal blood glucose levels is essential to ensure a good outcome. It is imperative to navigate this ideal carefully given the varying effects of pregnancy gestation on insulin sensitivity and the risk of iatrogenic hypoglycemia, especially if non-modified human insulin remains the mainstay of treatment. In 26% of our subjects, discontinuation of insulin therapy was associated with DKA events. The underlying reason for non-adherence was not determined. Diabetes-related knowledge, family support, fear of hypoglycemia, socioeconomic status, and insulin delivery devices have all been implicated as contributors to non-adherence (39). Based on our daily clinical practice, all of these factors, either individually or collectively, may contribute to non-adherence and insulin discontinuation, with hypoglycemia often cited by patients as a major reason for discontinuing insulin or diverting from the prescribed insulin regimen.

In pregnant women with diabetes and imminent preterm delivery, betamethasone therapy enhances fetal lung maturation but also causes hyperglycemia and even DKA. Betamethasone was identified as a precipitant in 20% of events in this study, a significantly higher percentage compared to the seven percent noted in Rochester, Minnesota (37). Rigorous implementation of glycemic monitoring and appropriate correction of hyperglycemia are of paramount importance to prevent DKA events in these women (40).

In the index study, a near quarter of women were diagnosed with diabetes at the time of the DKA event. This figure is significantly higher than the 12% of women who were undiagnosed in Rochester, Minnesota (37). Due to limited resources and access to health care locally, many pregnant women enter formal heath care for the first time during pregnancy. Further, although universal screening of glucose homeostasis during pregnancy would be ideal, our public health sector currently lacks the resources to do. Selective screening is therefore practiced in many regions within South Africa including the Western Cape, where the modified NICE criteria are used (41). The majority of women in our study were not tested timeously, despite having risk factors that dictated selective screening. Based on BMI alone, more than two thirds of our cohort qualified for an OGTT at 24 weeks to screen for GDM (41, 42). Our data, albeit limited, indicate that antenatal selective screening of glucose homeostasis is not implemented optimally, supporting the opinion that screening practices in South Africa remain limited (6, 43). In addition, less than half of the cohort with known diabetes underwent trimester-specific HbA1c measurements to aid antenatal glucose monitoring. A high median HbA1c of 10.4 (7.7-11.9) in pregnant mothers at the time of the DKA event, indicates poor glycemic control in those with pre-existing as well as newly diagnosed diabetes. Both undiagnosed diabetes and poor glycemic control are very likely to have enhanced the risk of DKA events in our patient cohort, irrespective of the identified precipitating causes.

There was a high prevalence of spontaneous fetal demise in our cohort, with 23% of the cohort losing their baby. These results concur with historic reports (9). As most of the spontaneous intrauterine deaths occurred at the time of the DKA, the hypothesis that the DKA contributed significantly is supported. However, to what extent maternal acidosis, dehydration with reduced uteroplacental perfusion, electrolyte imbalance per se or a combination of these factors contributed, remains unclear. Prior studies have shown that the risk of stillbirth increases with the severity of maternal acidosis (10, 32, 33, 44), a finding that we were unable to confirm. According to the study, fetal demise rates are higher in pregnancies complicated by DKA than in more recent studies in developed countries such as the United States and the United Kingdom (32, 33). An earlier local study conducted in Pretoria, South Africa, found an even higher rate of fetal death (31%) despite similar levels of acidosis (35). Every year an estimated 2.6 million stillbirths occur worldwide, with up to 98% in low and middle-income countries (45, 46). Stillbirth has multifactorial etiologies. Increased maternal age and BMI are established risk factors, whereas hypertension and diabetes, especially if poorly controlled, are the most common maternal conditions known to contribute (45–47). As stillbirths occur more frequently in low and middle income countries, low socio-economic status and poor access to formal health care are regarded as significant contributors (45).. Many of these established contributors were highly prevalent in our study population but appeared similar between pregnancies with live births and those with fetal loss. The small numbers in our study, however, precluded us from drawing firm statistical conclusions.

In our study, hypokalemia and hypoglycemia, which may result in arrhythmias, respiratory failure, and fetal fatalities, were common consequences of DKA treatment (48–50). Hypokalemia was shown to be significantly associated with fetal loss. (p=0.04) Five women who experienced hypoglycemia and fetal loss had concurrent hypokalemia, suggesting a compound risk that requires further examination in a larger study population.

The prevalence of obesity amongst South African women of childbearing age is concerning and escalating (35.2% from 24.7% in 1998) (51). Over two-thirds (68%) of our cohort were either overweight or obese. The mechanism that underlies the observed association between obesity and stillbirth remains elusive and multifactorial. Obese women are more likely to develop gestational hypertension and diabetes and is associated with an increased risk of apnoeic-hypoxic events, as well as the development of uteroplacental insufficiency earlier in pregnancy (52, 53). A study conducted in sub-Saharan Africa found that obese Zimbabwean women had a seven fold increased risk of preeclampsia and a fivefold increase in T2D (OR 5.41.91.5% CI 1-27.0) (54).

Diabetes is one of the major causes of stillbirths worldwide. A significant correlation exists between the metabolic control of the mother during pregnancy and the adverse outcomes for hyperglycemic pregnancies (55). Uncontrolled diabetes in early gestation, especially if present in the first few weeks, adversely impacts placental growth and development and predispose to intra-uterine growth restriction (56–58). Hyperglycemia causes endothelial dysfunction, which contributes to the development of hypertensive conditions such as pre-eclampsia during pregnancy (47, 59). Both these conditions are known to contribute to the risk of fetal demise irrespective of hyperglycemia. Pre-DKA HbA1c values were unacceptable for both live-births and fetal losses in this cohort, indicating suboptimal diabetes management and compliance. As we had uniform uncontrolled hyperglycemia, we were unable to determine the degree to which hyperglycemia itself contributed to perinatal mortality. Discordant fetal growth in the setting of diabetes in pregnancy is also associated with fetal demise. In a study from the Western Cape, South Africa an increased risk for stillbirth was noted for small for gestational age pregnancies between 33- and 40-weeks’ gestation and for large for gestational age babies after 37 weeks (50). The most common type of fetal growth abnormality seen in diabetic pregnancies is large for gestational age (LGA) babies or macrosomia (baby’s birth weight exceeding 90th percentile or 4000 grams) (60). Maternal overweight, the degree of maternal hyperglycemia, gestational weight gain, and maternal lipids all contribute to fetal overgrowth (61). In contrast, poorly controlled diabetes may result in intrauterine growth restriction due to suboptimal placental development if present in early gestation and may also result in growth restriction if associated with established microvascular disease (56, 57). In our study, maternal glucose control was poor, yet only a single baby was classified as macrosomic. While gestational age could be a confounder, hyperglycemia in women with pre-existing diabetes may have caused placental insufficiency rather than excessive growth and macrosomia in our study.

The clinical course of diabetes types is known to vary greatly, and there is evidence that some patients with adult-onset diabetes share characteristics of both T1D and T2D (20). T1D is, however, considered to be an autoimmune disease caused by autoantibodies against pancreatic β-cells (62). Anti-GAD antibodies are biomarkers of T1D-associated autoimmunity that can be used to identify and study patients at risk of developing T1D in advance of the disease’s onset. Approximately 1-10% of people with T2D and up to 38% of women with GDM have anti-GAD antibodies (63–67). The presence of anti-GAD antibodies has also been shown to be predictive of the onset of postpartum diabetes in women with GDM (63). Despite reports that genotypes differ according to ethnicity, Padoa found no ethnic difference between black Africans and white persons with T1D in South Africa (67). Anti-GAD antibodies were tested in twenty-one women without known T1D at the time of DKA in our study. These included 14 with HFDP and seven women with a prior diagnosis of T2D. The result of the anti-GAD test was positive in six of the tested women (29%). In light of these findings, we believe that pregnant women with DKA should be strongly considered for auto-immune diabetes regardless of their BMI or prior classification as T2D. Knowledge of anti-GAD antibody status in this subset of pregnant mothers may be particularly useful to determine insulin dependency or in identifying women who might benefit from emerging treatments for the prevention of T1D.

In our background reproductive female population, we have a heavy metabolic footprint and obesity is a specific concern. There is some controversy as to whether obesity trends in the general population are indicative of obesity rates in people with T1D, or whether they are related by genetics and environmental susceptibilities (68, 69). Nearly half of women with T1D who had a DKA event in pregnancy in our study were overweight or obese, thus warning against reliance on clinical phenotype to dictate diabetes subtyping. The findings of this study are consistent with recent findings from other studies that indicate that obesity is a highly prevalent problem in individuals with T1D (68–71). According to Evertsen et al., current T1D patients have higher BMIs than their predecessors and are also younger at disease onset (71).

Hypertensive disorders of pregnancy account for 10-24% of all stillbirths among low- and middle-income countries (45, 72). Studies reporting on hypertensive disorders in pregnancy affected by DKA’s are limited. Most women in our cohort had some form of hypertension. Forty-seven percent of women had pregestational hypertension and gestational hypertension was documented in a further 26%. In addition, suspected pre-eclampsia (either superimposed or *de novo*) complicated 36% of pregnancies. Dhanasekaran noted hypertension in 17% of their cohort in Minnesota (37). Another study of pregnant mothers with T1D from Boston also documented a high percentage (34%) of hypertensive disorders in pregnancies affected by DKA. Pregestational hypertension accounted for 15%, gestational hypertension 7% and preeclampsia 12% of cases (10, 37). Surprisingly neither hypertension nor pre-eclampsia were present in any of their pregnancies with fetal loss (10). This is in contrast with our work where most IUDs occurred in women with hypertension (6/11, 55%).

DKAs in pregnancy have rarely been studied in detail at the patient level. Due to the retrospective and descriptive nature of this study, causality cannot be inferred, however, it contributes to the current knowledge of pregnancy outcomes and complications associated with DKA. The small sample size prohibited robust statistical analysis. Furthermore, neither the specific socioeconomic status of the participants nor their competency in managing their diabetes were formally assessed.

The high rates of obesity and hypertensive disorders, as well as suboptimal antenatal glycemic control, potentially contributed to the high number of intrauterine deaths observed. Significant implementation gaps remain in screening for hyperglycemia and antenatal diabetes care in resource-constrained environments. These gaps must be eliminated if dangerous complications like DKA are to be minimized (42). Clinicians should strive to ensure continuous development and implementation of strategies that ensure optimal preconception and antenatal management of diabetes, as well as empowering women with diabetes through education. Unintended iatrogenic consequences of DKA management such as hypokalemia and hypoglycemia should be minimized with strict protocols to limit the possibility of fetal loss. In the context of high-risk populations for DKA and healthcare providers, we emphasize the importance of ongoing structured diabetes education. Through the use of these data, local practices can develop targeted protocols and interventions that can decrease the risks associated with hypokalemia, ultimately improving patient outcomes and inspiring a culture of continuous improvement.

## Conclusion

This study found a high risk of fetal mortality and undiagnosed auto-immune diabetes in women with DKA during pregnancy. There was a strong correlation between hypokalemia and fetal loss, suggesting a window of opportunity for addressing management gaps.

## Data availability statement

The datasets presented in this study can be found in online repositories. The names of the repository/repositories and accession number(s) can be found below: https://figshare.com/s/ced95d7e93e95fa0d5ef.

## Ethics statement

Informed consent was waived due to the retrospective nature of data collection. The study complied with the World Medical Association Declaration of Helsinki. It was approved by the Health Research Ethics Committee (HREC) of the Faculty of Medicine and Health Sciences, Stellenbosch University (S21/11/255). The studies were conducted in accordance with the local legislation and institutional requirements. The ethics committee/institutional review board waived the requirement of written informed consent for participation from the participants or the participants’ legal guardians/next of kin due to the retrospective nature of data collection.

## Author contributions

AC: Conceptualization, Data curation, Investigation, Project administration, Writing – original draft, Writing – review & editing, Methodology. DH: Conceptualization, Methodology, Supervision, Writing – review & editing. MV: Formal Analysis, Software, Writing – review & editing. EL: Writing – review & editing. MC: Data curation, Formal Analysis, Investigation, Methodology, Supervision, Writing – review & editing.
